# Supplementary material for: Mindfulness supports emotional resilience in children during the COVID-19 pandemic
Source: PLoS One. 2023 Jul 12;18(7):e0278501. doi: 10.1371/journal.pone.0278501 (PMC10337965; doi:10.1371/journal.pone.0278501)
Supplement: S1 File — (DOCX) [file pone.0278501.s001.docx]

**Supporting Information**

# S1 Text. *Recruitment and Inclusion Criteria.*

We analyzed the pre-intervention (baseline) data from children and their parents in a remote reading intervention study, collected from July 2020, through February 2021. Third and fourth-grade children across the United States were recruited through Facebook ads, schools, flyers, and word of mouth. We aimed to recruit children from lower SES backgrounds and therefore reached out to school districts with high percentages of students eligible for free/reduced lunch and targeted Facebook ads to lower income zip codes across the country. Since a number of schools that we reached out to had significant numbers of Spanish-speaking families we provided parents with the opportunity to indicate whether Spanish was their preferred language for communication. The research team included bilingual staff, fluent in Spanish, who translated all written parent communication and assessments into Spanish, and were available to communicate in Spanish with parents who indicated that was their preferred language.

Interested parents filled out an eligibility screen and were invited to participate in a baseline session (Pretest 1) if initial inclusionary criteria were met (child speaks English proficiently, has normal or corrected-normal hearing, has access to a computer or tablet at home, and has a parent that speaks English or Spanish). Further inclusionary criteria required that the child achieve a standard score of 80 or above on the Kaufman Brief Intelligence Test (KBIT) Matrices subtest, a standardized nonverbal IQ assessment (Kaufman & Kaufman, 2004a).

#

# S2 Text. *COVID-Specific measure.*

CHILD COVID IMPACT Scale

1. How much have you been bothered by COVID-19?

2. How much harder has it been to see your friends because of COVID-19?
2b. If they answer anything besides “not at all” ask: How much do you miss seeing your friends?

3. How much harder has it been to see certain family members because of COVID-19?
3b. If they answer anything besides “not at all” ask: How much do you miss seeing those family members?

4. How much has COVID-19 changed the kinds of activities you get to do?
4b. If they answer anything besides “not at all” ask: How much have those changes in activities bothered you?

* 2b, 3b, & 4b are drop down questions which only get asked if the answer to the prior question is something besides ‘not at all.’

---------------------------------------------------------------------------------

# S3 Text. *Measure validation: CFA*.

As we constructed our own measures of negative affect and child COVID-impact, we conducted a post-hoc confirmatory factor analysis in *lavaan* to identify possible limitations in those measures (Rosseel 2012). We examined the internal validity of the constructed scales of child COVID-Impact and child negative affect. The Negative Affect scale, derived from two proposed factors on thirteen affect items, was upheld. The seven negative affect items had comparable loadings, and the fit was reasonable (CFI=0.91, RMSEA=0.083). However, the one-factor loading of the Child COVID-Impact scale showed an imperfect fit (CFI=0.876, RMSEA=0.306).

**S1 Fig.** Most subjects were from high income households. Total parental income is plotted by frequency of participants. Ten subjects chose not to respond.

**
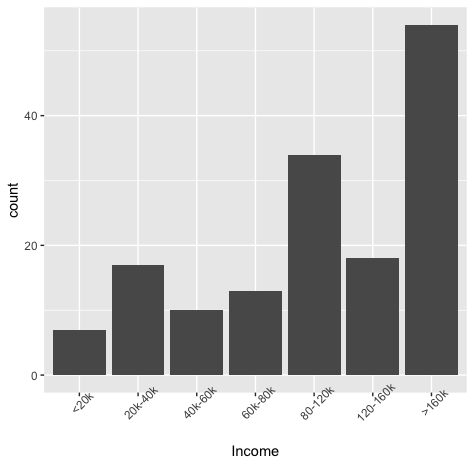
**

**S2 Fig.** Most subjects were from families with high maternal education. Maternal education is plotted by frequency of participants. Eight subjects chose not to respond.

**
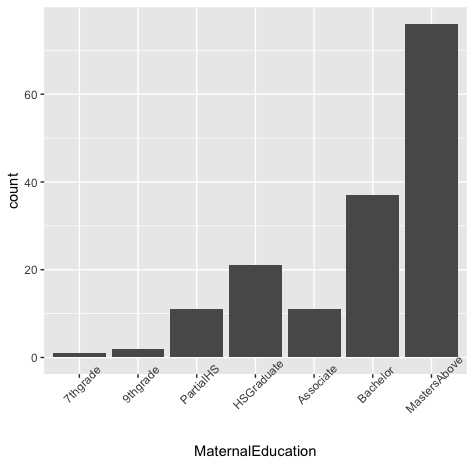
**

**S3 Fig.** Prior mindfulness experience. We asked caregivers whether their children had any previous experience with mindfulness. Results are shown separated for children with ADHD, with anxiety, with ADHD and anxiety, and with no diagnosis of ADHD or anxiety.


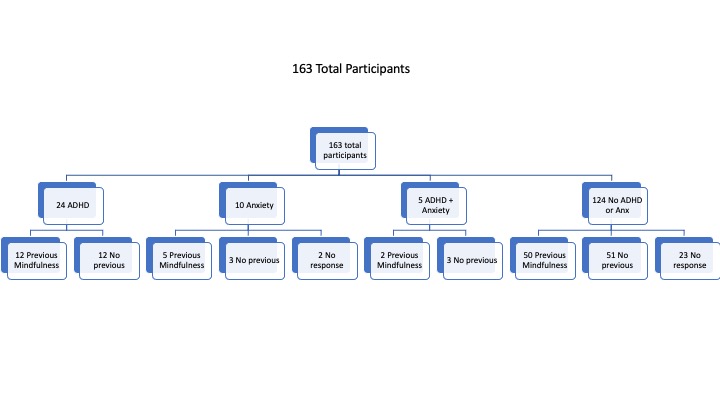


**S1 Table.** CAMM is not a significant moderator of the relationship between Child COVID-Impact and RCADS – anxiety.

| **Model: Anxiety** | Estimate | Standard Error | t value | Pr(>\|t\|) |  |
| --- | --- | --- | --- | --- | --- |
| (Intercept) | 41.874 | 0.751 | 55.789 | 0.0000 | *** |
| covidimpact | 0.816 | 0.546 | 1.493 | 0.1379 |  |
| camm_score | -3.405 | 0.549 | -6.205 | 0.0000 | *** |
| Gender | -1.917 | 1.051 | -1.824 | 0.0705 | . |
| Age | -0.680 | 0.537 | -1.264 | 0.2084 |  |
| MaternalEducation | 0.269 | 0.522 | 0.514 | 0.6079 |  |
| covidimpact:camm_score | -0.825 | 0.523 | -1.577 | 0.1172 |  |
| *Signif. codes: 0 <= '***' < 0.001 < '**' < 0.01 < '*' < 0.05 < '.' < 0.1 < '' < 1* | | | | | |
|  | | | | | |

**S2 Table.** CAMM is not a significant moderator of the relationship between Child COVID-Impact and RCADS – depression.

| **Model: Depression** | Estimate | Standard Error | t value | Pr(>\|t\|) |  |
| --- | --- | --- | --- | --- | --- |
| (Intercept) | 44.664 | 0.887 | 50.373 | 0.0000 | *** |
| covidimpact | -0.469 | 0.649 | -0.722 | 0.4713 |  |
| camm_score | -4.261 | 0.648 | -6.571 | 0.0000 | *** |
| Gender | -2.263 | 1.247 | -1.815 | 0.0718 | . |
| Age | -0.191 | 0.638 | -0.299 | 0.7657 |  |
| MaternalEducation | 0.235 | 0.621 | 0.378 | 0.7060 |  |
| covidimpact:camm_score | -0.825 | 0.618 | -1.336 | 0.1841 |  |
| *Signif. codes: 0 <= '***' < 0.001 < '**' < 0.01 < '*' < 0.05 < '.' < 0.1 < '' < 1* | | | | | |
|  | | | | | |

**S3 Table.** CAMM is not a significant moderator of the relationship between Child COVID-Impact and PSS-C.

| **Model: Stress** | Estimate | Standard Error | t value | Pr(>\|t\|) |  |
| --- | --- | --- | --- | --- | --- |
| (Intercept) | -0.037 | 0.116 | -0.319 | 0.7503 |  |
| covidimpact | -0.052 | 0.084 | -0.614 | 0.5404 |  |
| camm_score | -0.411 | 0.087 | -4.726 | 0.0000 | *** |
| Gender | -0.021 | 0.164 | -0.126 | 0.8995 |  |
| Age | -0.094 | 0.083 | -1.132 | 0.2599 |  |
| MaternalEducation | -0.059 | 0.081 | -0.730 | 0.4670 |  |
| covidimpact:camm_score | -0.070 | 0.083 | -0.841 | 0.4022 |  |
| *Signif. codes: 0 <= '***' < 0.001 < '**' < 0.01 < '*' < 0.05 < '.' < 0.1 < '' < 1* | | | | | |
|  | | | | | |

**References**

Kaufman, A.S., & Kaufman, N.L. (2004a). Kaufman Assessment Battery for Children, Second

Edition. Bloomington, MN: Pearson, Inc.

Rosseel, Y. (2012). lavaan: An R package for structural equation modeling. *Journal of Statistical*

*Software*, *48(2),* 1-36.
